# Vascular graft infection due to *Pasteurella multocida*

**DOI:** 10.1186/s40064-015-1638-7

**Published:** 2015-12-30

**Authors:** F. Fourreau, F. Méchaï, J. Brossier, O. Bouchaud, B. Picard

**Affiliations:** Service de médecine interne, CHU Kremlin Bicêtre/Assistance publique-hôpitaux de Paris et Université Paris, Sud 78, rue du générale Leclerc, Le Kremlin Bicetre, 94270 France; Service de Bactériologie-Virologie, Hygiène, CHU Avicenne/Assistance publique-hôpitaux de Paris et Université Paris 13, 125, rue de Stalingrad, Bobigny, 93000 France; Service des maladies infectieuses et tropicales, CHU Avicenne/Assistance publique-hôpitaux de Paris et Université Paris 13, 125, rue de Stalingrad, Bobigny, 93000 France; Service de chirurgie vasculaire et thoracique, CHU Avicenne/Assistance publique-hôpitaux de Paris et Université Paris 13, 125, rue de Stalingrad, Bobigny, 93000 France

**Keywords:** *Pasteurella multocida*, Graft infection, Vascular graft

## Abstract

**Background:**

Vascular graft infections are infrequent complications with important morbidity and mortality rates. *Pasteurella multocida*, a Gram negative bacillus, is a normal oral commensal of many animals. For mankind, it is a pathogenous bacillus which is rarely implicated in vascular grafts.

**Case report:**

We report hereafter the fourth case introduced in the international literature about vascular graft infections caused by *P. multocida*. The patient was successfully treated with a combination of a surgical graft change and a 6 weeks bi-antibiotic therapy.

**Discussion:**

There is fours case reported in litterature with quite different antibiotic drugs and duration.

**Conclusion:**

*P. multicoda* graft infection should be long with initial intravenous drug and mainteance traitement should not be required.

## Background

The most common clinical infection of *Pasteurella multocida* is a local cellulitis subsequent to animal scratches or bite wounds. The second pathological form is a pulmonary localization and affects persons close to animals (Hubbert and Rosen 1970).

The incidence of bacterial vascular graft infection is estimated between 0.5–5 % depending of the graft localization, according to a 5 years follow up (Goldstone and Bowersox 1996). The infection appears 7 months after surgery with a limb’s amputation risk from 10 to 70 % and a mortality rate from 10 to 30 % (Bandyk 2000; Hennes et al. 1996). The most frequently isolated germs are *Staphylococcus aureus* (30 %) and *Staphylococcus epidermidis* (17 %). We noticed here an infection of *P. multocida* vascular graft after an asymptomatic cat scratch.

## Case report

A 59 years old Caucasian woman was admitted in December 2011 to the emergency department of our institution for a tumefaction in the right scarpa. This painful and fast expending pulsatile tumefaction was developed over a week and was accompanied by fever at 39–40 ℃.

Her case history indicated a left iliofemoral bypass in 2005 and a changed by femoral left right crossover graft in April 2009. Moreover a tobacco addiction and an alcohol intoxication were stopped in 2006 with neither cirrhosis nor chronic obstructive pulmonary disease. In 2008, she had a T2N2aM0 tonsil cancer, treated by surgery and radiotherapy. She has been considered in remission since 2008.

**Fig. 1:**
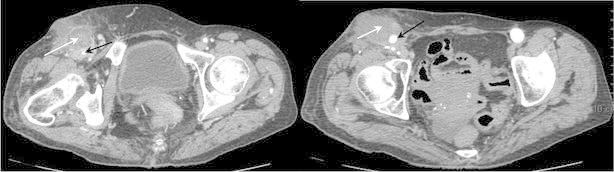
Abcess of right scarpa on contrast enhanced computer tomography scan. *White arrow* abcess. *Black arrow* graft

In the emergency department, the clinical examination showed an abscess next to the right scarpa. Pertinent laboratory data on admission included the following polymorphonuclear leukocyte count: 19.550 G/L (normal range: 1.5–7 G/L) and chain reactive protein (CRP) at 211 mg/L (upper normal: <10 mg/L). Other biological exams were normal. The contrast enhanced computer tomography scan of the pelvis and leg showed an abscess contracted with the vascular graft without sign of septic perforation (Fig. 1). The following day, the patient showed an active skin bleeding due to an abscess fistula with a graft perforation. She was operated in emergency, with a flattening of the abscess, a removal of the bypass distal part replaced by an impregnated silver salt Dacron (Intergard Silver, Maquet, Rastatt, Germany) allowing the revascularization of the profunda femoris and sartorius plasty. The culture of intraoperative samples from the vascular graft and the abcess pus both yield *P. multocida*. All other samples (blood cultures and fluid suction drains) remained sterile. In vitro culture indicated that the strain was sensitive to penicillin, third generation cephalosporin, aminoglycosides, cotrimoxazole, fluoroquinolones and fosfomycin. The initial empiric treatment was instituted pre-operative by piperacillin/tazobactam (4 g/500 mg × 4 per day) combined with amikacin (15 mg/kg once a day). It was modified 2 days after according to the bacteriological results by ofloxacine 200 mg × 3 per day combined with cotrimoxazole (800/160 mg) 1cp × 3 per day for 6 weeks. After 7 days of this antibiotic treatment, the patient had no fever anymore and a clean scar without pain or inflammatory signs. From a biological standpoint, there was a regression of hyperleukocytosis (7600 G/L) and a decrease in CRP (54 mg/L). Fifteen days after the surgery, the patient was admitted into a convalescent center. Four weeks after the introduction of antibiotic therapy, she presented a scar division of right scarpa without sign of infection and a good vascularization of the distal lower limb. Laboratory data was returned to normal. Three mouths after surgery, she has still the scar division without any infectious sign.

## Discussion

Pasteurella are small Gram negative coccobacilli with bipolar and aerobic-anaerobic respiratory type. These bacteria are able to induce a severe inflammatory reaction and necrotizing lesions probably due to a lipopolysaccharide endotoxin production. *P. multocida* has a natural resistance to vancomycine and clindamycin but remains sensitive to many antibiotics: β-lactams, fluoroquinolones, tetracyclines and imidazole. Macrolides and aminoglycosides have a lower activity. The β-lactams and tetracyclines—alone or combined with imidazoles–macrolides or aminoglycosides are the antibiotics advised to treat pasteurellosis and infections after bites or scratchs. Doxycycline–bacteriostatic antibiotic—is used sparingly in severe infections, particularly bacteremia. Therefor and in reason of their good pharmacological and pharmacokinetic data, fluoroquinolones are often favored. In vascular graft infection, the choice of the mono or bi therapy antibiotic is not really defined.

Pasteurella are found on the mucous membranes of the mammal and bird aerodigestive tract (Weber et al. 1984). Four species are responsible for most human infections (*P. multocida, P. canis, P. stomati* and *P. dagmatis*), but *P. multocida* is the most frequently isolated in human pathology. Human infection from bites, scratches or licking damaged skins has mainly a feline (60–80 % of cases) or canine origin (90 % of remaining cases) (Weber et al. 1984) and comes exceptionally from plant stings. The infection rate reported after medical consultation for cat or dog bites is between 7 and 17 %. *Pasteurella* colonization and infections at the respiratory tract’s level, with or without bite or scratch are described among people who have frequent contacts with animals (i.e. veterinarians, owners of small animals and farmers) (Hubbert and Rosen 1970; Jones and Smull 1973).

In human pathology, *P. multocida* is responsible for several types of infections. It causes abscesses and cellulitis ducts by direct bite inoculation. They are characterized by a short incubation of several hours with a very sore inflammatory pain and the appearance of lymphangitis during the second day. The infection may be spontaneously cured or progress to a subacute form in the absence of antibiotics treatment. Subacute forms appear several weeks after inoculation and are characterized by a reactive arthritis and tenosynovitis with important functional sequelae. There are also blood-borne infections, with significant mortality on immunocompromised patients (specially cirrhosis) (Weber et al. 1984; Stein et al. 1983) where *P. multocida* has been implicated in lung abscesses, meningitis, brain abscess, but also a few cases of endocarditis. This species can also be grafted on foreign implants. There are several cases reported in the literature mainly orthopedic prosthetic infections but only three published cases are reported for vascular prosthesis infections (Kalish and Sands 1983; Sannella et al. 1987; Kessler et al. 2004).

In our case, it appeared that the patient owned a cat. She received numerous scratches on exposed parts. During the month before the infectious episode, the patient did not notice a localized cutaneous inflammatory reaction or antibiotics use. The study of three cases of vascular graft infection with *P. multocida* reported in the literature associated with ours (Table 1) shows that the case of Kalish et al. (1983) can be classified in early infections with a period of 2 months after surgery, unlike the other three patients with a period between six and 32 months. Three patients had undergone a graft removal for a vascular complication including a false aneurysm 2 months before the infectious episode for Kalish et al. (1983) and an occlusion of the iliac arteries 32 months before the infection for our patient. Contamination was evident for two cases [dog licking a toe’s stump only 2 weeks after the amputation for Kalish et al. (1983) and cat bites treated with amoxicillin/clavulanic acid 15 days before the infection for Kessler et al. (2004)]. In the case reported by Sannella et al. (1987) as well as our patient, no deep or symptomatic lesions or direct animal contact with the wound were found. However, in both cases, patients explained having received numerous scratches or bites without local or systemic consequences in the weeks before their hospitalization. The clinical presentation of the four patients showed mainly a major pain in the groin (Kalish and Sands 1983; Kessler et al. 2004) associated, in the case reported by Sannela et al. (1987), with intermittent claudication. The other relevant clinical element, for our patient and the ones of Kalish et al. (1983) and Kessler et al. (2004), was a pulsatile mass with a quick increase in size. In the case reported by Kalish et al. (1983), the toe scars were cleaned during the graft infection. The four patients were managed by partially or totally removing the infected prosthesis: a combination of intravenously β-lactam antibiotics for a period between 19 days and 6 weeks, followed by ampicillin for Kalish et al. (1983) for several months and doxycycline for lifetime for Kessler et al. (2004).

**Table 1:** Main characteristics of the three cases of graft infection in *P. Multicoda* reported in litterature and our case

	Kalish et al. (1983)	Sannella et al. (1987)	Kessler et al. (2004)	Patient reported in our observation
Age (years)	61	78	73	59
Infected graft localisation	Femoral crossover bypass	Aortobifemoral bypass	Aortobifemoral bypass	Femoral crossover bypass
Time of infection after prosthetic restoration (months)	24	30	6	32
Contamination’s source	Via a wound	Asymptomatic scratch	Symptomatic scratch	Asymptomatic scratch
Symptoms	Fever pain erythema pulses’ abolition	Flow	Painpulsatile mass	Fever pain pulsatile massquick increase
Antibiotic therapy	Ampicilline	Cefalotine	Ampicilline then ceftriaxone	Ofloxacine/ cotrimoxazole
Duration of antibiotic therapy	19 days	3 weeks	6 weeks	6 weeks
Maintenance therapy	Several months^a^	no	Doxycycline for life time	no

^a^Duration of maintenance therapy unspecified in the publication

Surgery: Partial removal of a graft portion in contact with the abscess, leaving hardware in place. Total: removal and replacement of the entire vascular equipment

In this case and the one with Sannela et al. (1987), no deep lesion was found either next to the scar or remotely. This situation could tend to suspect symptomatic or asymptomatic bacteremia, with risk of graft infection in patients with vascular prosthesis. Few cases are reported in the literature but when patients are bitten and scratched by animals, prophylactic antibiotics could be discussed for patients with prosthetic material to prevent secondary bacterial graft.

## Conclusion

When scratch or animal bites are identified, a bacterial graft infection should be suspected with *P. multocida* no matter where they are from the scar or material. Then, the antibiotic treatment, such as other bacterial prosthetic infections, should be quite long—mainly 6 weeks—with initial intravenous drug. However the maintenance therapy should not be required. Finally, as few cases have been reported, the use of primary prophylaxis is not significant for all people including those who have animals or prosthesis.
